# Imaging the vasculature of a beating heart by dynamic speckle: the challenge of a quasiperiodic motion

**DOI:** 10.1117/1.JBO.28.4.046007

**Published:** 2023-04-25

**Authors:** Aurélien Plyer, Elise Colin, Xavier Orlik, Ali Akamkam, Julien Guihaire

**Affiliations:** a Université Paris Saclay, DTIS, Onera, Palaiseau, France; b ITAE Medical Research, Péchabou, France; c Université de Toulouse, Onera /DOTA, Toulouse, France; d Université Paris-Saclay, Unité de Recherche Préclinique, Hôpital Marie Lannelongue, Groupe Hospitalier Paris Saint Joseph, Le Plessis-Robinson, France

**Keywords:** dynamic speckle, speckle contrast imaging, cardiology, cardiac imaging, optical flow

## Abstract

The spatial and temporal evolution of the field backscattered by a beating heart while illuminated with a coherent light reveals its macro- and microvascularization in real time. To perform these vascularization images, we use a recently published method of laser speckle imaging, based on the selective detection of spatially depolarized speckle field that is mainly generated by multiple scattering. We consider the calculation of the speckle contrast, by a spatial or temporal estimation. We show that the signal-to-noise ratio of the observed vascular structure can be noticeably increased by a postprocessing method implying the calculation of a motion field that allows the selection of similar frames extracted from different heartbeat periods. This later optimization reveals vascular microstructures with a spatial resolution of the order of 100  μm.

## 1 Introduction

*In vivo* characterization of heart diseases benefits from various imaging techniques that use the properties of ultrasounds, x-rays, radioactivity, and magnetic resonance. They exhibit complementary advantages and the ability to reconstruct a volumetric image. The noninvasive technique of three-dimensional (3D) echocardiography is particularly suited for real-time imaging of the cardiac volume chambers and valves and is a powerful tool for evaluating congenital abnormalities. X-rays are routinely applied for imaging of coronary artery disease (CAD), either by invasive coronary angiography (ICA) or by coronary computed tomography angiography.^1^ Radioactivity is also used in the case of cardiac scintigraphy for myocardial perfusion imaging.^2^ Magnetic resonance imaging provides noninvasive anatomical and functional features, including millimeter-scale analysis of cardiac valves, ventricular volumes, and myocardial perfusion.^3^ Donors with extended criteria (advanced age, and cardiovascular comorbidities) are routinely allocated for heart transplantation nowadays. Severe CAD is a major determinant of primary graft failure and early death after transplantation. ICA is recommended for diagnosis of CAD in organ donors older than 55 years or in those older than 45 years with cardiovascular risk factors. However, ICA is only performed in one-third of donors at risk for CAD, mainly due to logistic reasons. *Ex situ* heart perfusion (ESHP) is an innovative approach to improve the preservation of the donor heart before transplantation.^4^^,^^5^ This method allows for continuous organ monitoring including metabolic assessment of myocardial viability. ICA is the gold standard method for CAD diagnosis because of its high contrast and spatially resolved imaging capabilities but has been poorly reported during ESHP because of potential iatrogenic concerns including myocardial edema due to accumulation of contrast agent, coronary dissection, and infection.^6^^,^^7^ Dang Van et al. recently reported their preliminary experience of *ex situ* perfusion of the donor heart in high risk transplantation.^4^ In this clinical series, surgeons performed a coronary angiography during ESHP. This procedure resulted in a major myocardial edema and primary graft failure after transplant. The authors stated that contrast agent used for *ex situ* coronary imaging was deleterious for the heart in this setting. There is, therefore, a need for an alternative safe coronary imaging protocol to discriminate donor hearts with CAD that would not be eligible for transplantation.

We sought to apply an alternative noninvasive approach to explore the coronary circulation on the donor heart before transplantation. The intention of this work is to develop a method that in the future could be utilized to explore the coronary circulation of donor hearts. Here, we propose a complementary tool based on the light scattering, to assess the structure and functionality of the cardiac vasculature. We test it in this paper for a healthy heart. As a full-field optical technique, it allows high-resolution imaging of the entire peripheral vasculature of the heart with real-time imaging capabilities. The proposed technique is based on a previously published paper that presents the laser speckle orthogonal contrast imaging (LSOCI) method developed to selectively detect multiple scattering of moving red blood cells.^8^

The speckle phenomenon results from the summation of waves backscattered by a rough surface or object when illuminated by a coherent source, such as a laser. Coupled with a camera, the dynamic speckle techniques designate the recording and the exploitation of a temporal sequence of a speckle pattern, useful when the scatterers undergo a significant movement during the integration time. Indeed, during the integration of the photons by the camera, if the scatterers are in movement, this generates a blurring phenomenon of the speckle, which can be detected in the statistical characteristics of the intensity distribution. The main parameter to see this statistical change is called the speckle contrast coefficient. It is defined as the coefficient of variation, that is, the ratio between the standard deviation and the average value of the intensity distribution in each pixel. Indeed, the coherent backscattered light from moving particles leads, when observed with an adequate integration time, to a blurring effect of the detected optical interference thus to a decrease of its temporal or spatial contrast. This technology, called LSCI for laser speckle contrast imaging,^9^^,^^10^ is based on the pioneering work of Fercher and Briers.^11^

However, the full potential of this technique can still be upgraded and especially from the point of view of polarimetry. In fact, we previously analyzed with high accuracy the polarimetric properties of experimental speckles at the spatial scale of their correlation length.^12^^,^^13^ The various polarimetric properties of the observed speckles were found to be very dependent on the scattering origin. Indeed, while surface scattering mainly conserved the illumination state of polarization, multiple scattering that occurs generally deeper in volumes did spread the polarimetric states all over the Poincare sphere.^14^ These observations lead us to propose a polarimetric upgrade of the LSCI method called LSOCI.^8^ The principle consists of detecting the speckle backscattered by the moving particles using a polarimetric filtering. The latter selects mainly the photons that undergo multiple scattering under the surface, by suppressing the photons from the first-order scattering, which mainly preserve the polarimetric state of the laser illumination. Thus, this polarimetric filtering has to project the backscattered speckle field onto a polarimetric state orthogonal to the one of illumination.

Since in this configuration, the speckle decorrelation time at each pixel is mainly driven by volume scattering, the measured parameter was called volume microvascular activity index, and defined by

$$\mathrm{VMAI}=\frac{1}{TC_{⊥}^{2}},$$ (1)

where T is the integration time of the camera, and $C_{⊥}$ is the speckle contrast detected in the orthogonal polarimetric configuration. $C_{⊥}$ is estimated on populations of several samples, as the ratio of the standard deviation on the mean.

In this paper, we propose to adapt an LSOCI system to obtain the peripheral microvasculature of a cardiac muscle. As the imaging process is performed during the rapid movement of a beating heart, this represents a challenge for all dynamic speckle-based techniques such as LSOCI. Indeed, the key criterion of speckle contrast can be estimated in a purely spatial way, from a single image, from

$$C_{⊥}(x)=\frac{\sqrt{\frac{1}{N}\underset{x_{p}\in \omega (x)}{\sum }{\left(I(x_{p})-\overset{¯}{I(x)}\right)}^{2}}}{\overset{¯}{I(x)}},$$ (2)

where the symbol $\overset{¯}{.}$ denotes the mean value over spatial position, ω(x) denotes a spatial neighborhood of pixel x, and N is the number of pixels in this neighborhood.

However, these spatial estimates are at the expense of resolution. This loss of resolution is particularly damaging if one wishes to preserve the restitution of the finest microvessels. Therefore, the temporal estimation of the contrast coefficient is an attractive alternative for estimating the VMAI index. It is defined as

$$C_{⊥}(x)=\frac{\sqrt{\frac{1}{N}\overset{N}{\underset{k=1}{\sum }}{\left(I_{k}(x)-\langle I(x)\rangle \right)}^{2}}}{\left\langle I(x)\right\rangle },$$ (3)

where $I_{k}(x)$ is the value of the pixel x in the image number k, ⟨.⟩ denotes the temporal average, ⟨I(x)⟩ is the mean value of the images along time: $\left\langle I(x)\rangle =1/N\overset{N}{\underset{k=1}{\sum }}I_{k}(x\right)$, and N is the number of images used to compute the contrast.

But in this case, it is necessary to question the robustness of the estimation toward a nondesired motion of the object and the influence of the acquisition rate on the estimates. Indeed, Ref. 15 revealed the difficulty and the importance of compensating the overall movements of the imaged object before a reliable exploitation, even with the use of a high-speed camera.

In this article, we highlight the possibility of obtaining images of peripheral vasculature of the heart using a dynamic speckle system, by investigating the different possibilities of spatial and or temporal estimations of the speckle contrast. After the presentation of a process to visualize the vasculature in real-time using spatial contrast, we propose a postprocessing method based on the estimation of a displacement field between acquired frames that takes advantage of the periodic movement of the heart to enhance the signal-to-noise ratio of the LSOCI signal. We call this method multiperiod enhanced signal-to-noise ratio (MPE-SNR). Then, we demonstrate the ability of the MPE-SNR method to significantly improve the imaging capability of LSOCI, by revealing microvessels as small as 100  μm despite the heart movement.

## 2 Materials and Methods

**Fig. 1:**
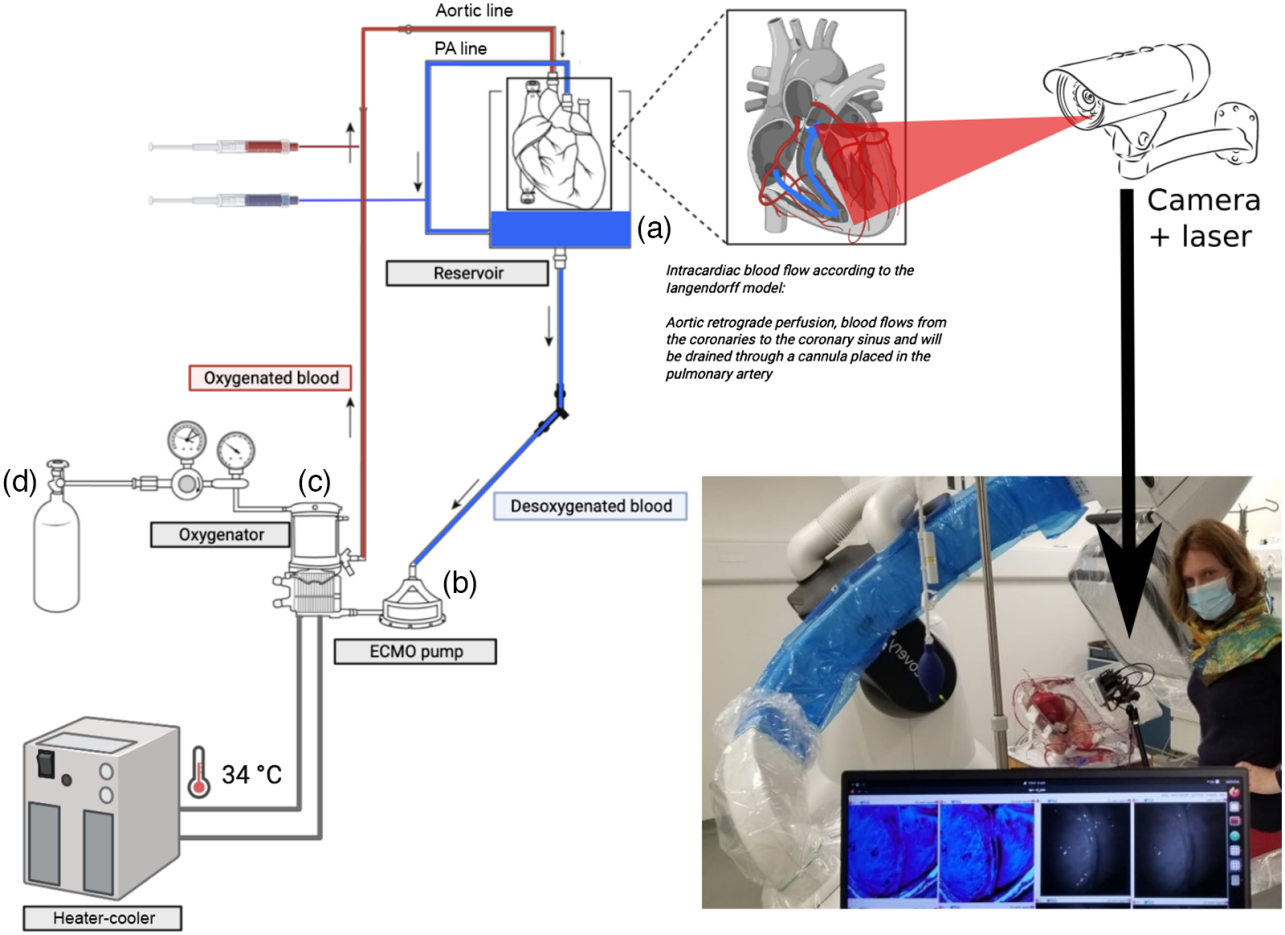
Experimental platform for isolated ESHP using oxygenated warm blood. The heart is perfused in (a) a dedicated module connected to a circuit including (b) a centrifugal pump, (c) a membrane oxygenator with (d) gas exchanger and a heater-cooler. PA, pulmonary artery.

This study is part of the ECHORONEX project dedicated to *ex vivo* imaging of the donor heart before transplantation. Porcine hearts were retrieved in large white piglets (60 kg) after infusion of a cold crystalloid cardioplegia solution in the aortic root (Del Nido solution, 1 L). All hearts were instrumented for isolated *ex vivo* perfusion on a dedicated perfusion module (Organ Care System, TransMedics, Andover).^16^ All hearts were perfused at 34°C with porcine oxygenated blood and paced at 80 beats per minute using bipolar epicardial electrodes connected to an external pacemakers as previously described in Ref. 17 (Fig. 1). The experimental protocol was approved by the Institutional Committee on Animal Welfare (APAFIS #35023-2022012717164093 v4, Animals Ethics Committee of the University of Paris Saclay, France). Animals were treated in accordance to the “Guidelines for the Care and Use of Laboratory Animals” developed by the National Institute of Health and with the “Principles of Laboratory Animal Care” developed by the National Society for Medical Research.

**Fig. 2:**
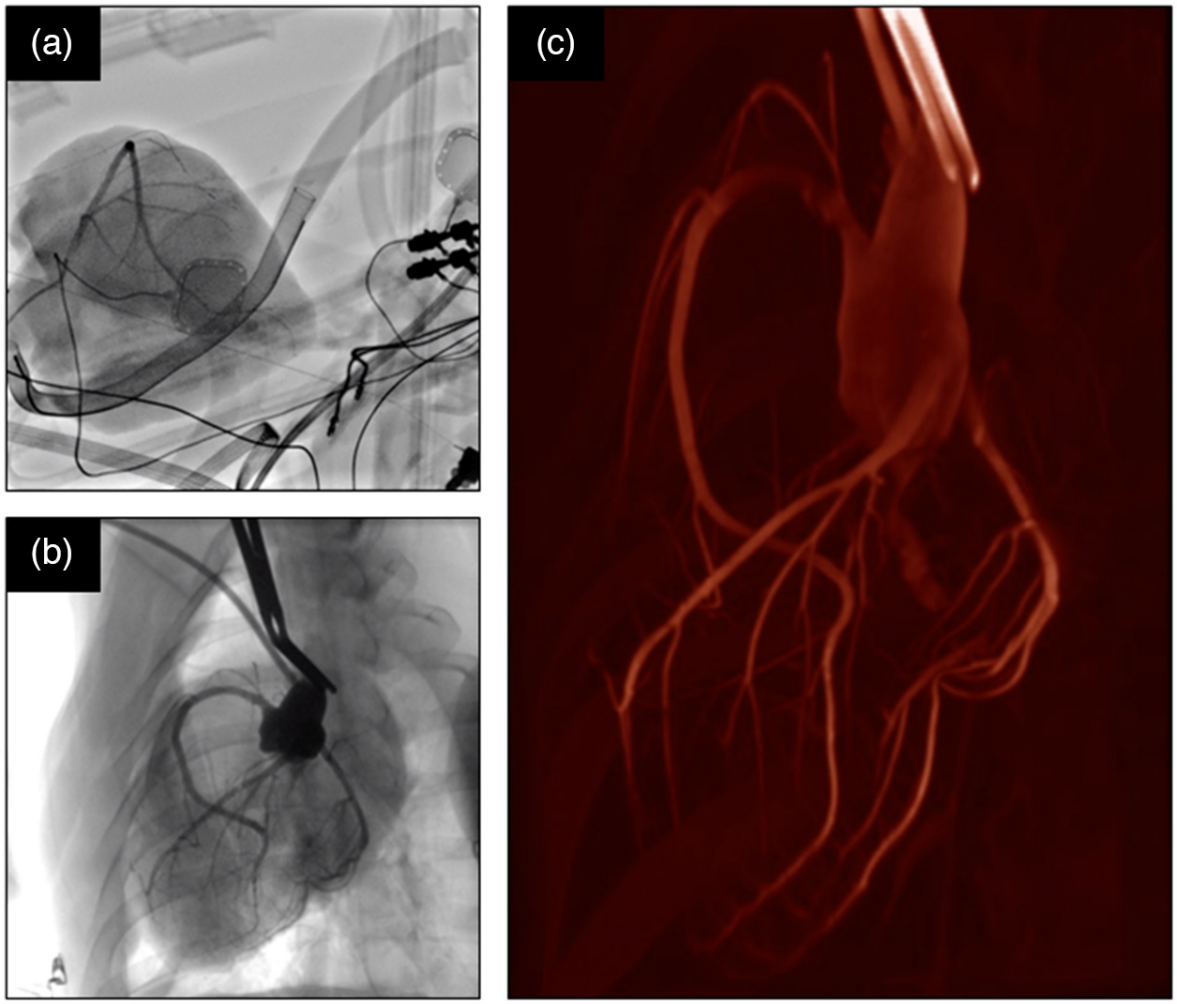
Anatomy reference images: (a) coronary angiography and (b) cardiac CT scan imaging obtained for the first pig, (c) 3D reconstruction from the CT scan.

Coronary angiography and coronary computed tomography angiography are gold standard methods for coronary artery imaging in clinical practice. To investigate the anatomical distribution of the coronary arteries in our experimental porcine model, we performed a coronary angiography and a coronary computed tomography angiography in the first pig of the present study, as shown in Fig. 2. We observed a normal distribution including a left main coronary artery and a right coronary artery. We then assumed that this anatomy was similar among all our study subjects since they come from the same siblings.

For the LSOCI experimental part, we use a laser Lambda Mini from RGB Photonics, with a wavelength of 785 nm and a power of up to 70 mW. The sensor is a polarimetric camera (Sony MX250), equipped with a four-directional polarization square pixel array which can filter light from all four directions in a single capture. The frame rate is 102 fps for a field of view described by 800×800  pixels, and we set the integration time T to 2 ms after a qualitative optimization.

The system is mounted on an articulated arm, which can be fixed directly above the operating table to image the heart just before its extraction, or on the support above the perfusion module. The acquisitions are made for two consecutive minutes.

All the hearts in the present experimental protocol were paced at 80 bpm, to ensure the reproducible management and assessment of these hearts. LSOCI acquisitions can be triggered at any desired time during the experiment. They do not require synchronization with the electrical activity of the heart, and thus, there is no slaving needed between the OCS perfusion system and the speckle acquisition.

## 3 Real-Time Visualization by Using Spatial Contrast

First, we compute the spatial contrasts of each video frame corresponding to the polarization orthogonal to the laser one. Indeed, since the movement of the heart is complex and fast, it is not possible to use a temporal contrast calculation on consecutive frames.

To improve the quality of the spatial contrast image obtained while maintaining a computational speed compatible with real-time, we use an infinite impulse response filter combining two consecutive images. Thus, for each iteration i, we warp the contrast map from the previous iteration i−1 according to the estimated flow between the frames. The estimated spatial contrast $C_{s}^{i}$ estimated according to Eq. (2) is then replaced by its filtered version C^si, following the equation:

C^si(x)=αCsi(x)+(1−α)C^si−1(x+wii−1(x)), (4)

where α is a fixed coefficient, $w_{i}^{i-1}$ is the average movement between the contrast images i−1 and i, x is the spatial position within the image. In the following, α is set to 0.3.

The movement is computed by a dense optical flow algorithm, called EFolki.^18^ This algorithm relates to the estimation of a dense motion, i.e., in each pixel, with a local regularization constraint. It is, therefore, perfectly suited to the calculation of a motion that does not present discontinuity, and that is not rigid, since the heart deforms throughout the sequence. The interest of this algorithm among those of optical flows is that it has been optimized from the point of view of the computation speed/robustness compromise.

**Fig. 3:**
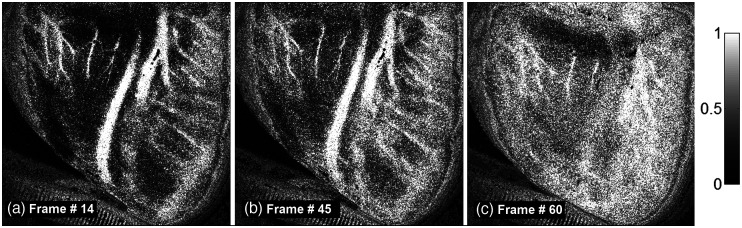
Three 1/C^si images, obtained from spatial contrast, extracted from a cardiac period. (a)–(c) Frames #14, #45, #60 within a reference cycle, with increasing estimated heart motion.

The images of the Fig. 3 are video frames extracted from the video made in real-time on the heart, with the spatial contrast method. We extract three frames at different moments of the cardiac cycle: the first one when the position of the heart stabilizes, the others during the beating movement. In this figure and thereafter for the activity images, we display the inverse of the squared contrast parameter, since it is directly proportional to the VMAI in Hertz. At this stage, this parameter 1/C^s was simply thresholded between two constant values for all our acquisitions, fixed at the first and 99th percentile of the statistical distribution of the values recorded during the first acquisition. Then, the parameter is simply normalized between 0 and 1 thereafter for a common display in the following.

These first images allow us to visualize, in real-time, some structures of the vasculature. The video for the cardiac cycle obtained in real-time is given in Fig. 11, Video 1.

## 4 Multiperiod-Enhanced Signal-to-Noise Ratio Method

In the case of *a posteriori* processing, we propose to take advantage of the periodic movement of the heart to enhance the signal-to-noise ratio of the LSOCI signal.

First, we focus on the construction of a video sequence representing a reference cardiac cycle. Each contrast frame of this sequence is optimized using the sequences of the whole recording, by taking advantage of temporal redundancies at pulse periods. Then, we combine the images from this sequence to produce a single image of vasculature.

### 4.1 Video for a Single Cardiac Cycle

We know that to improve the signal-to-noise ratio of a speckle image, we need to increase the number N of realizations involved in our estimates.

Therefore, we start by considering a reference sequence of $n_{T}$ successive images representing the duration of a cardiac cycle, that is, $n_{T}=[f/f_{B}]$ where $f_{B}$ is the heartbeat rate converted in standardized frequency in Hertz ($f_{B}=80/60$), and f is the camera’s image frequency. ⌈.⌉ denotes the ceiling function.

For each of the k image indices of this reference sequence, we compute what we subsequently call a k-bucket, i.e., the subset of the indices of the images located around the cardiac repetition periods:

$$k\text{-bucket}=\underset{p\in I}{⋃}A_{k,p}\;\text{where }A_{k,p}=k+[\lceil p\frac{f}{f_{b}}-1\rceil ,\lfloor p\frac{f}{f_{b}}+1\rfloor ],$$ (5)

where the intervals $A_{k,p}$ contain two or three elements, depending on the existence or not of a decimal part for $p\frac{f}{f_{b}}$, respectively.

**Fig. 4:**
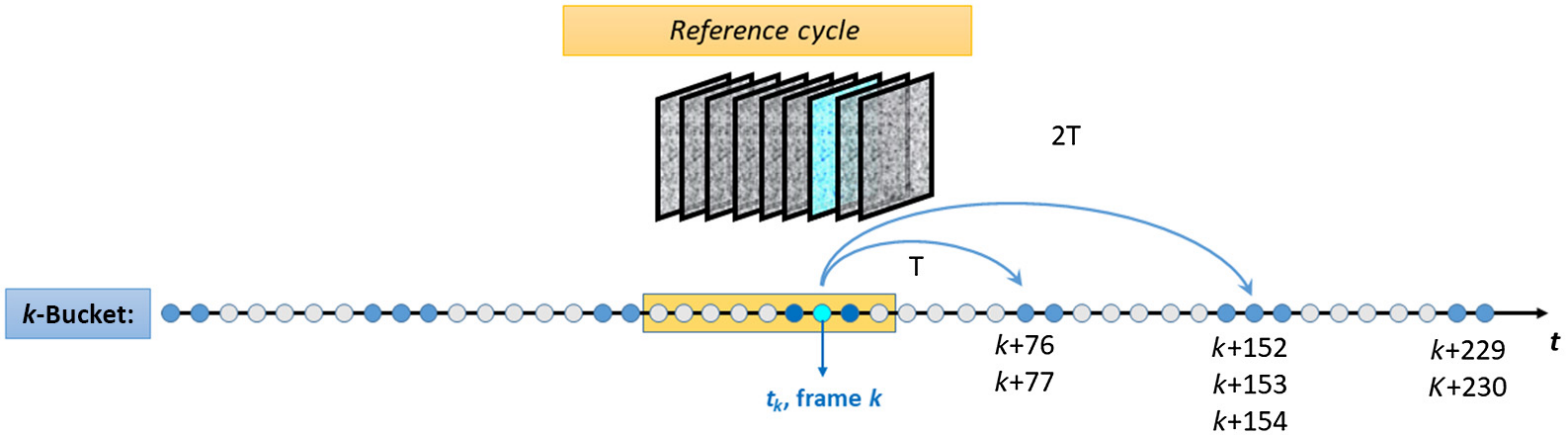
Schematic diagram of the selection of video frame pairs constituting a k-bucket, with $f_{B}=80/60\;\mathrm{Hz}$, f=102  Hz, $n_{T}=77$.

The schematic representation of this k-bucket is shown in Fig. 4. The main idea is to build a set of frames with similar heart positions in the picture.

In practice, the heart does not strictly follow a periodic motion at the period imposed by the perfusion machine. Sometimes, two successive images will be more similar to each other than two images separated by an exact number of cardiac periods. This is why we leave a latitude of selection of the images around the replicas with exact periods.

Afterward, to filter this k-bucket, we compute the set of motion fields between the spatial contrast of image k and the spatial contrast images for the set of images in the k-bucket:

$$F_{k}=\{\overset{¯}{w_{j}^{k}(x)},j\in k-\text{bucket}\},$$ (6)

where the symbol $\overset{¯}{.}$ denotes the mean value over spatial position. Finally, we consider the $N_{f}$ first indices of elements of $F_{k}$ in sorted order. This resulting set is called filtered k-bucket.

**Fig. 5:**
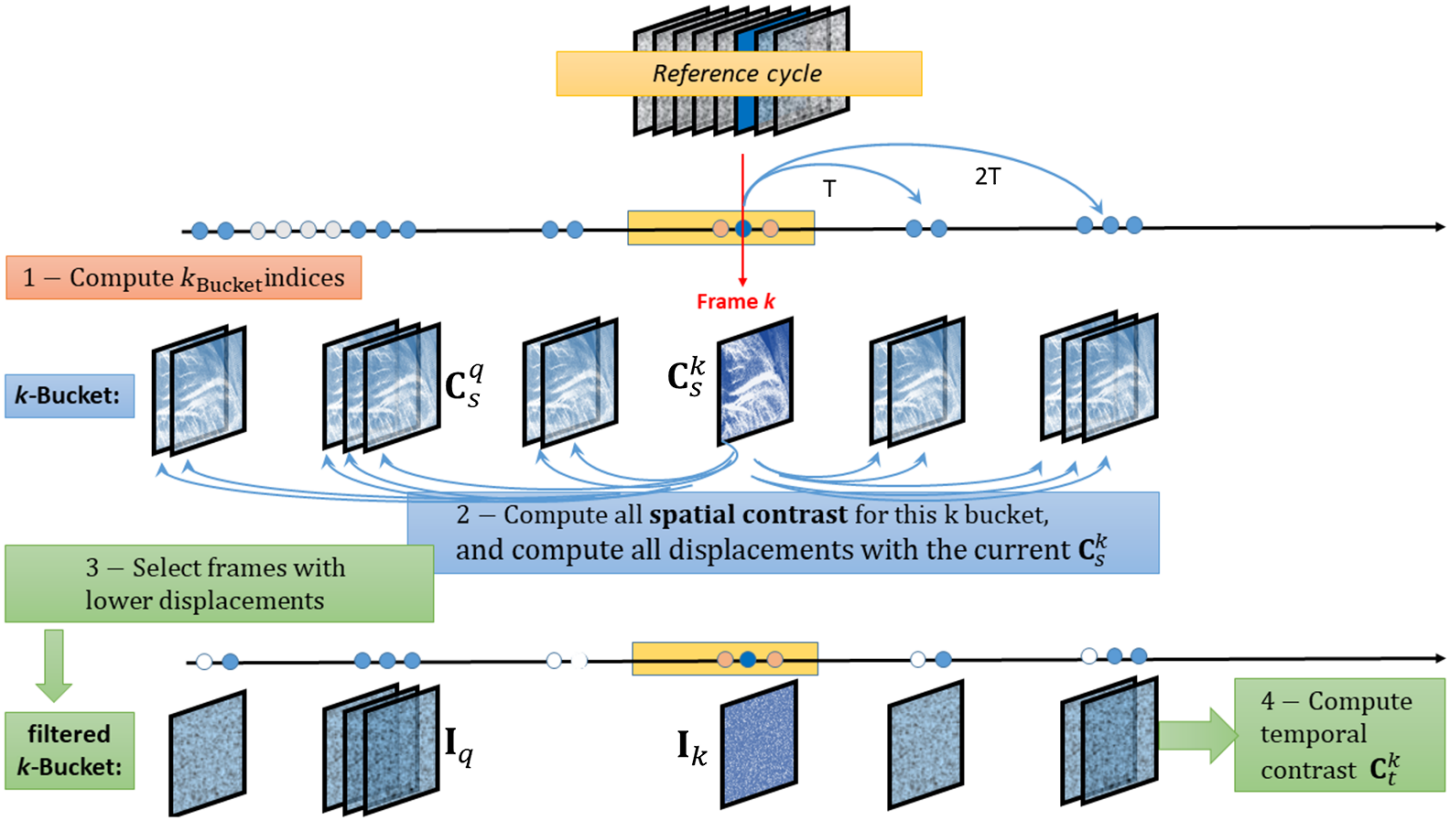
Explanatory diagram of the MPE-SNR optimization, for one frame extracted from the reference cardiac cycle.

For each index k of the reference period, a contrast image $C_{t}^{k}$ is computed temporally using the images whose indices belong to this filtered k-bucket, by:

$$C_{t}^{k}{\left(x\right)}^{2}=\frac{{\left\langle {I(x)}^{2}\right\rangle }_{\text{filtered }k\text{-bucket}}}{{\left\langle I(x)\right\rangle }_{\text{filtered }k\text{-bucket}}^{2}}-1,$$ (7)

where I(x) are the intensity images and ⟨.⟩ denotes the temporal average. Note that this estimate corresponds to the one of Eq. (3), in which the temporal estimation is on the indices of the k-bucket instead of on N indices of consecutive images. As the selected frames have small displacements between them, we do not need to resample them on the reference image. Figure 5 summarizes the steps to be performed to calculate the optimized image number k.

**Fig. 6:**
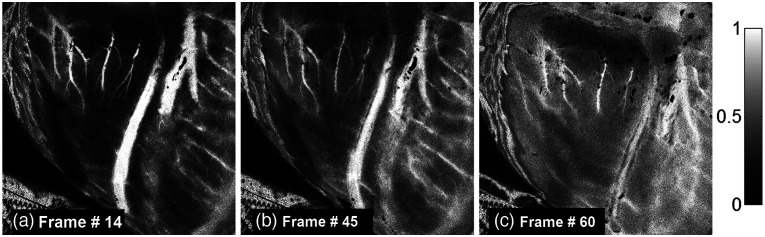
Three images of $1/C_{t}^{k}{\left(x\right)}^{2}$ obtained from the MPE-SNR optimization, extracted from the reference cardiac cycle. (a)–(c) Frames #14, #45, #60 within the reference cycle, with increasing estimated heart motion.

The video obtained on the central cardiac cycle is presented with the same reference frame selections as in the previous section. Resulting video frames are shown in Fig. 6.

The qualitative gain of the images obtained with our *a posteriori* optimization method can be observed by comparing these frames with those in Fig. 3. We have intentionally chosen the same reference frame numbers. The video for the cardiac cycle reconstructed by the MPE-SNR method is given in Fig. 12, Video 2.

### 4.2 Optimal Frame for the Vasculature Characterization

In this last step of signal optimization, we start again from the previous video and focus on the frames around the moment when the movement is weakest, to reconstruct an optimal vasculature image.

To do this, we calculate the frames to frames average movements for the indexes of the reference period:

$$m_{i}=\overset{¯}{w_{i}^{i-1}(x)},\;i\in \text{reference period}.$$ (8)

**Fig. 7:**
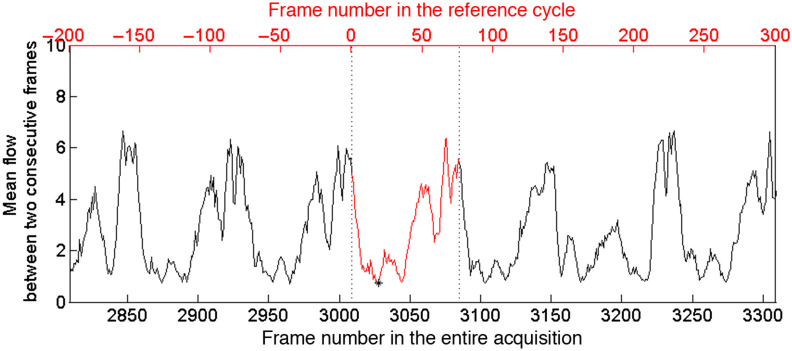
Example of a sequence $m_{i}$ of average optical flows calculated between iterative video frames of a heart reference period. The frame numbering axis for the sequence to be reconstructed is shown in red at the top of the figure. The frame numbering axis for the whole sequence is represented in black at the bottom of the figure. The sequence of optical flows $m_{i}$ passes a minimum for i=16.

Such a motion sequence $m_{i}$ is shown in Fig. 7. To realize the regularity of this pattern beyond the reference cycle, we calculated and represented it for the frames before and after the reconstructed sequence.

We select as a reference frame, the one for which the motion curve passes through a minimum. This frame is denoted as q. We then associate to it all the frames whose interframe motion is <1  pixel, and we compensate these video frames by calculating their displacement with the reference frame and rewrapping them in the reference frame of q. Then, we average the different contrast frames thus obtained as

$$C_{q}(x)={\left\langle C_{t}^{i}(x+w_{q}^{i}(x))\right\rangle }_{i:\overset{¯}{w_{i}^{i-1}(x)}<1}.$$ (9)

**Fig. 8:**
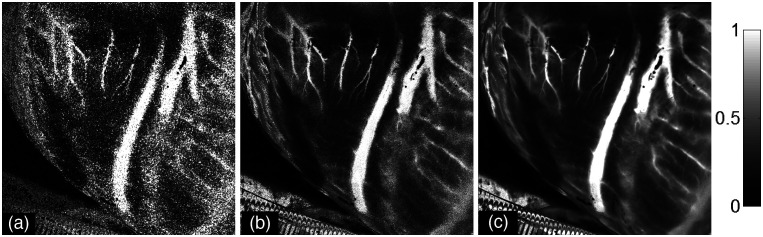
Comparison of reconstruction of isolated video frames: (a) 1/C^sq(x)2: isolated video frame of the real-time video based on spatial contrast; (b) $1/C_{t}^{q}{\left(x\right)}^{2}$: isolated video frame based of the MPE-SNR optimized temporal contrast; (c) $1/C_{q}{\left(x\right)}^{2}$: optimized frame based of the MPE-SNR optimized temporal contrast and motion compensation.

The best frame reconstruction obtained is shown in Fig. 8(c).

We first compare it in Fig. 8(a) to the image obtained for the same frame, by the first real-time procedure based on the filtered spatial contrast, without MPE-SNR. The first line is the whole image, while the second is a zoomed-in extract. Then, we show in Fig. 8(b) the reconstruction of the same frame based on the MPE-SNR process. Finally, Fig. 8(c) exhibits the MPE-SNR optimized by motion compensation. As expected, Fig. 8(c) shows the image with the lowest noise and reveals details in the vasculature.

**Fig. 9:**
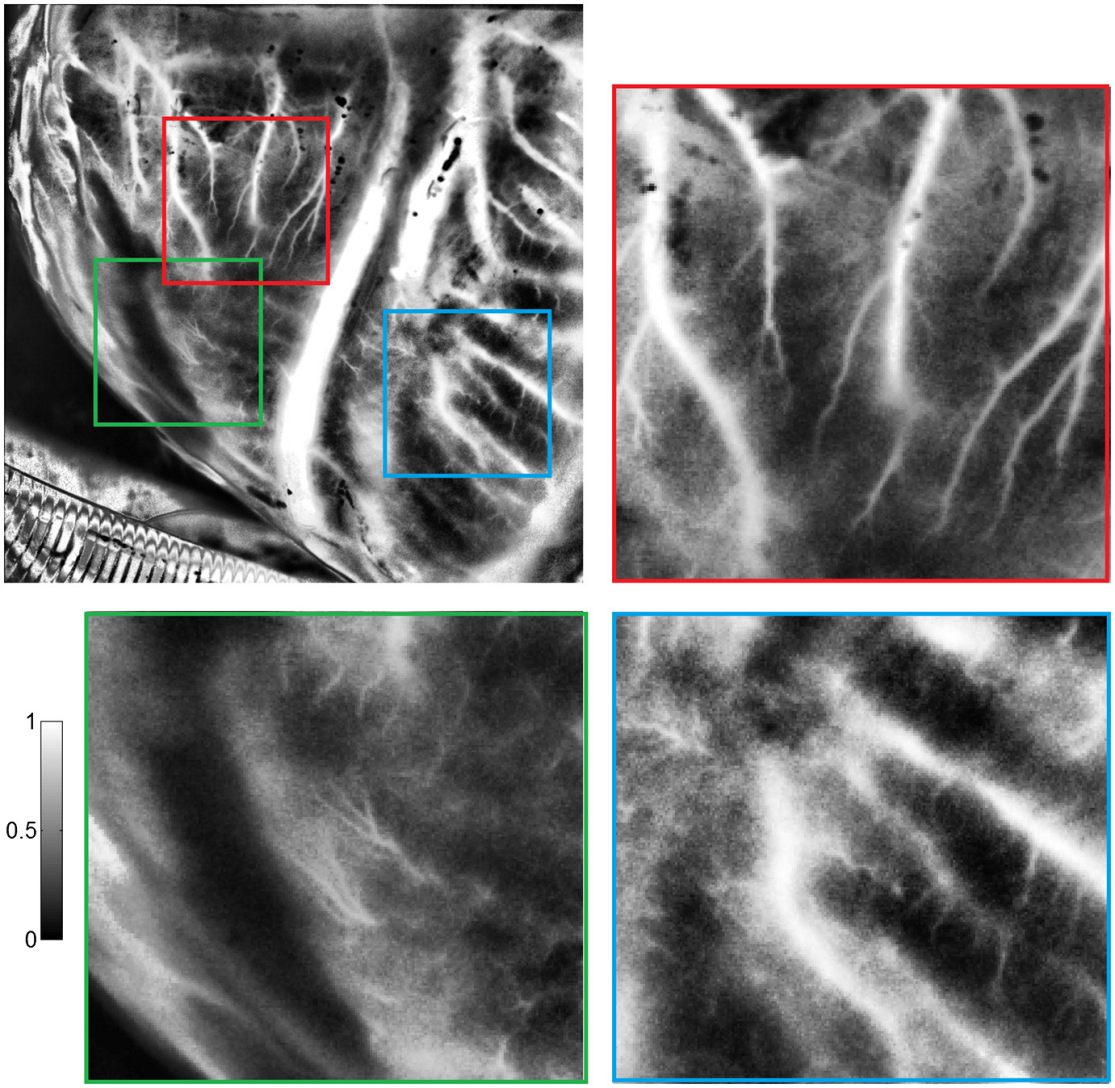
Three 200×200  pixel extracts from the optimal image $1/C_{q}^{2}$, after local histograph equalization.

The finest elements are perceptible at the pixel level. The contrast limited adaptive histograph equalization method in Ref. 19 makes them appear, as shown in Fig. 9.

## 5 Discussion

In the dimensioning of our acquisition, we preferred to use the highest possible acquisition frequency, to limit the effects of motion as much as possible. With this choice, the number of frames for an imposed heart period is not integer. We have seen that the consequences of this are minimal, indeed, the calculation of the flows between frames show us that it often happens that frames which are not separated by an integer number of periods are nevertheless closer than frames separated by an exact number of periods. This is certainly due to the fact that, even with an imposed rhythm, the movement keeps a nondeterministic part. In addition, the acquisition frequencies can drift in time. The ideal would therefore be to synchronize the two instruments, in addition to slightly modifying the frequencies so the number of frames per period is an integer.

The choice of the polarimetric camera allows us to see once again the gain of using cross-polarization. It is the one for which we obtain the best contrast images. In the future, we will try to optimize the use of the polarimetric information in each pixel, especially to compensate for possible 3D effects.

**Fig. 10:**
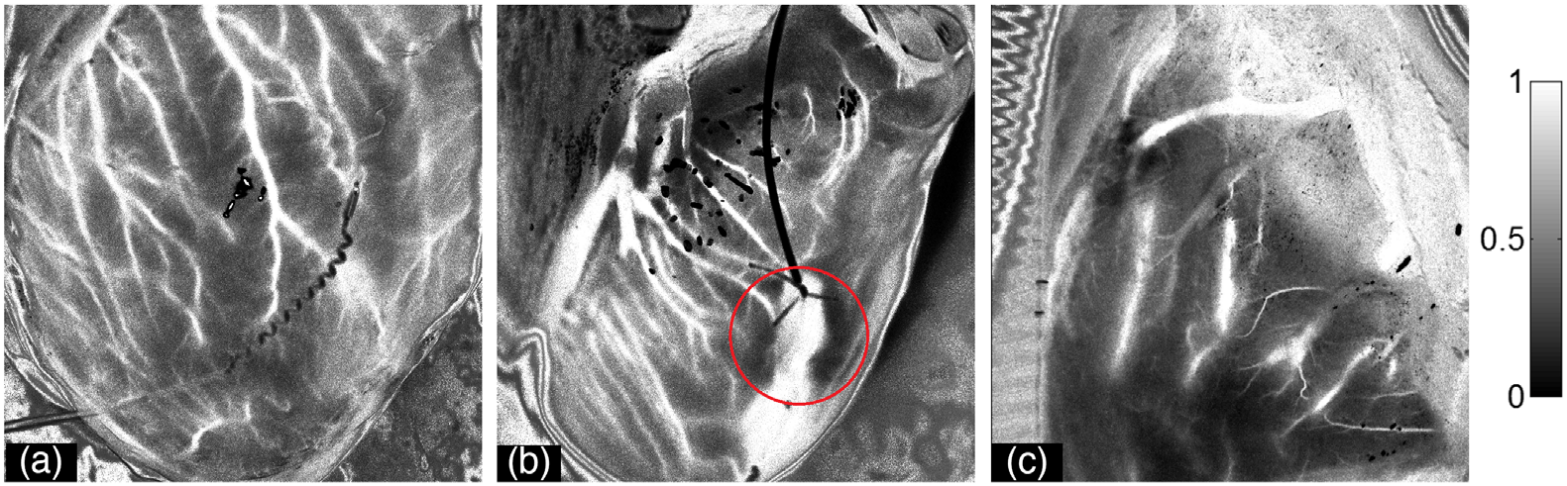
Three other temporal contrast images obtained on other pig hearts $1/C_{t}^{q}{\left(x\right)}^{2}$. In (b), a wound is circled in red. (a) Pig #4, (b) Pig #6, (c) Pig #7.

We also tested the robustness of the method by applying it to other acquisitions on various hearts. Figure 10 shows the variability of the results obtained. The quantification of the signal has not yet been implemented and is the subject of future works that will have to deal with both effects of the SNR-MPE method and 3D geometric curvatures on the microvascularization values. However, the variations of signal within the same image open the way to this future work. Figure 10(a) shows a different vascularization network. In Fig. 10(b), we have circled a wound in red. The very white spot corresponds to the visible blood flow around the pacemaker electrode; below it, a very dark spot, corresponds to a drop in the signal activity, probably due to a hematoma. Figure 10(c) corresponds to a shot from a different geometrical angle. It shows very fine vessels.

The longer term objective of this type of image is twofold. First, it is to help surgeons during operations to better visualize the coronary network in real-time, especially during coronary artery bypass grafting surgery. For this, online processing could be applied and investigated on *in vivo* heart data. Second and at a preliminary stage, we aim to evaluate the quality of myocardial perfusion during ESHP before the transplantation of a cardiac graft. We will then look for a correlation between currently available parameters measured during ESHP (coronary flow, mean aortic pressure, arterial, and venous lactates) and the activity index.

We hypothesize that early diagnosis of altered perfusion at the level of the coronary microcirculation would likely help prevent myocardial edema, a significant risk for primary graft failure after transplantation.

## 6 Conclusion

In this paper, we used a dynamic speckle method called LSOCI to image the micro- and macrovasculature of a beating heart in real time and in offline mode, taking advantage of the different recorded cardiac repeats. To do this, we combined spatial and temporal estimates of the contrast parameter.

The spatial speckle contrast is effective in visualizing the vasculature in real-time. Moreover, we show that it is possible to combine speckle realizations spaced by several periods, provided that we estimate the displacement of the heart between the different spatial contrast images recorded. This approach allows us to improve the signal-to-noise ratio and to observe both the macro- and microperipheral vasculature of the heart, despite its continuous motion. We have called this optimization method the MPE-SNR method.

The proposed LSOCI method coupled with the MPE-SNR allows imaging of the complete peripheral vasculature of a beating heart in a noninvasive way, with high spatial resolution and in only a few seconds. Moreover, the simplicity of the experimental setup should promote its intraoperative use for many surgical procedures. Future work is necessary for this imaging method, to compare flow measurements to those with ICA in a porcine model of CAD.

Although the transfer of the experimental protocol will raise many difficulties, it would be interesting to try to apply the method in clinical practice for *in vivo* evaluation. The method could also be used for the observation of other phenomena in the human arterial system that present periodic excitation of blood flow.

## 7 Appendix

**Fig. 11:**
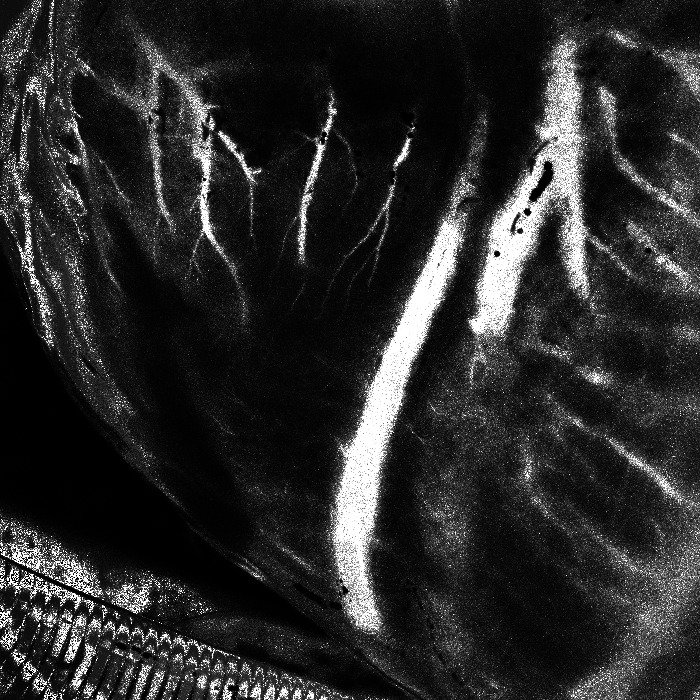
The real-time constructed video of the vasculature over a cardiac cycle (Video 1, MPEG, 8.7 MB [URL: https://doi.org/10.1117/1.JBO.28.4.046007.s1]).

**Fig. 12:**
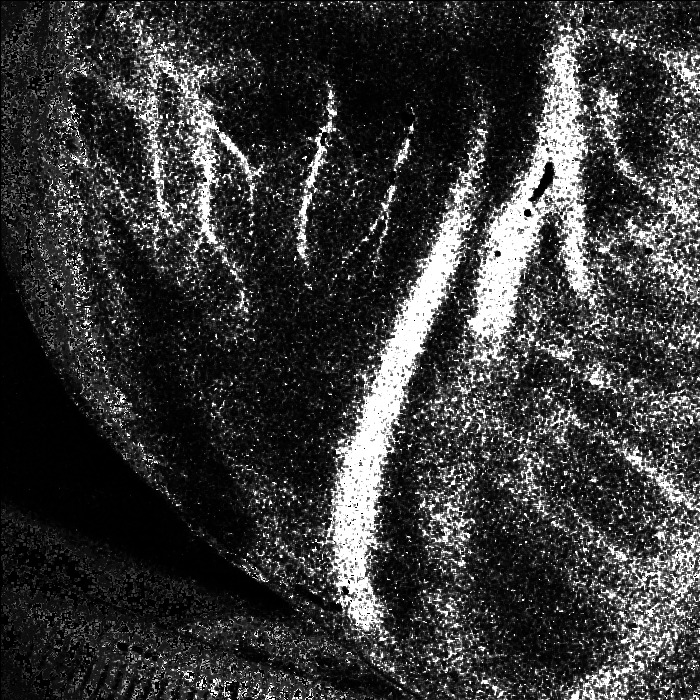
The offline constructed video of the vasculature over a cardiac cycle, using the MPE method (Video 2, MPEG, 5.6 MB [URL: https://doi.org/10.1117/1.JBO.28.4.046007.s2]).

Two video files are available: the first one in Fig. 11 contains a reconstruction of the vasculature over a cardiac period calculated in real time from the spatial contrast, and the second one in Fig. 12 contains a reconstruction of this same period calculated *a posteriori* using the MPE-SNR method.

## Code, Data, and Materials Availability

Data underlying the results presented in this paper are not publicly available at this time but may be obtained from the authors upon reasonable request.
